# Will climate change impact the potential distribution of a native vine (*Merremia peltata*) which is behaving invasively in the Pacific region?

**DOI:** 10.1002/ece3.1915

**Published:** 2016-01-11

**Authors:** Subhashni Taylor, Lalit Kumar

**Affiliations:** ^1^ Ecosystem ManagementSchool of Environmental and Rural ScienceUniversity of New EnglandArmidaleNSW2351Australia

**Keywords:** Biological invasion, climate change, CLIMEX, *Merremia peltata*, niche modelling, pacific region

## Abstract

*Merremia peltata* is a species with uncertain status in the island nations of the Pacific region. It has been designated introduced and invasive in some countries whereas it is considered native in others. Recent increase in its abundance across some island landscapes have led to calls for its designation as an invasive species of environmental concern with biological control being suggested as a control strategy. Climate change will add to the complications of managing this species since changes in climate will influence its range limits. In this study, we develop a process‐oriented niche model of *M. peltata* using CLIMEX to investigate the impacts of climate change on its potential distribution. Information on the climatic requirements of *M. peltata* and its current geographic distribution were used to calibrate the model. The results indicate that under current climate, 273,132 km^2^ of the land area in the region is climatically unsuitable or marginal for *M. peltata* whereas 664,524 km^2^ is suitable to highly suitable. Under current climate, areas of climatic suitability for *M. peltata* were identified on the archipelagos of Fiji, Papua New Guinea, Solomon Islands and Vanuatu. By the end of the century, some archipelagos like Fiji, Hawaii, New Caledonia and Vanuatu will probably become more suitable while PNG and Solomon Islands become less suitable for *M. peltata*. The results can be used to inform biosecurity planning, management and conservation strategies on islands.

## Introduction

Many biological invaders contribute to biodiversity loss by causing the extinction of native species (Sax et al. 2002). An invasive species is an introduced or non‐native species that becomes established and spread outside its native range (Sax et al. 2002). Invasive species are seen as one of the key drivers of change in island ecosystems and a major threat to native island biodiversity (Reaser et al. 2007; Ricciardi 2007). Island ecosystems are highly susceptible to biological invasions (Mueller‐Dombois and Fosberg 1998). The Pacific region includes three of the 35 global biodiversity hotspots (East Melanesian Islands, New Caledonia and Polynesia‐Micronesia) with numerous endemic and native species (Myers et al. 2000). Habitat destruction and invasive species are reported as the two main causes of species' extinctions in this region (Sherley et al. 2000). These non‐native species often have highly detrimental impacts on native biota, leading to alterations at the community, the ecosystem, and the landscape levels (Vitousek et al. 1996).

**Figure 1:**
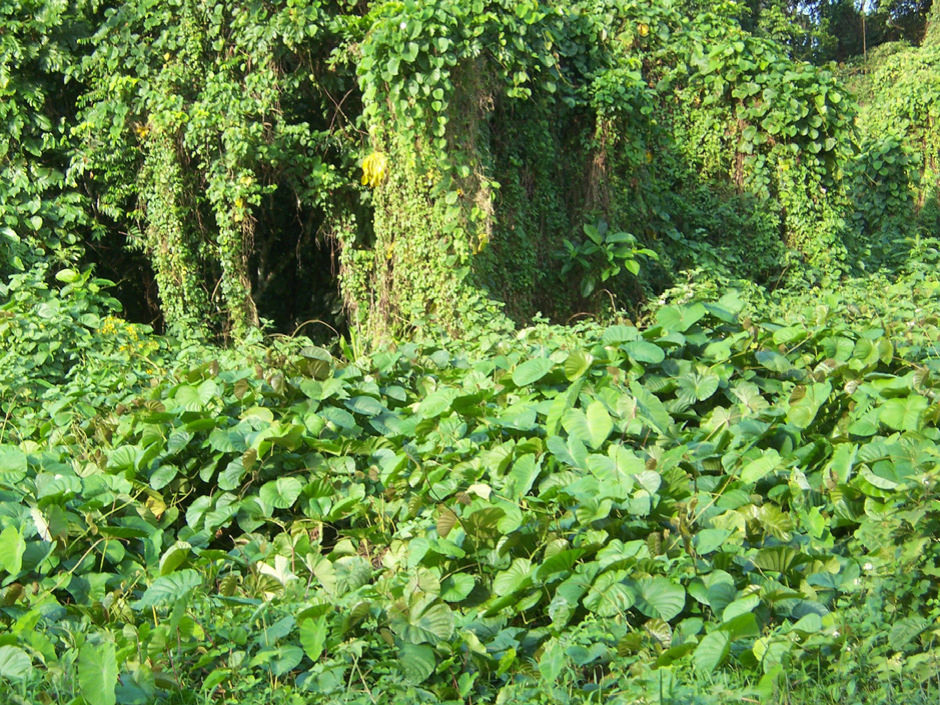
Photograph of *Merremia peltata* on a roadside on the island of Espiritu Santo, Vanuatu. Photo credit: Subhashni Taylor.

**Figure 2:**
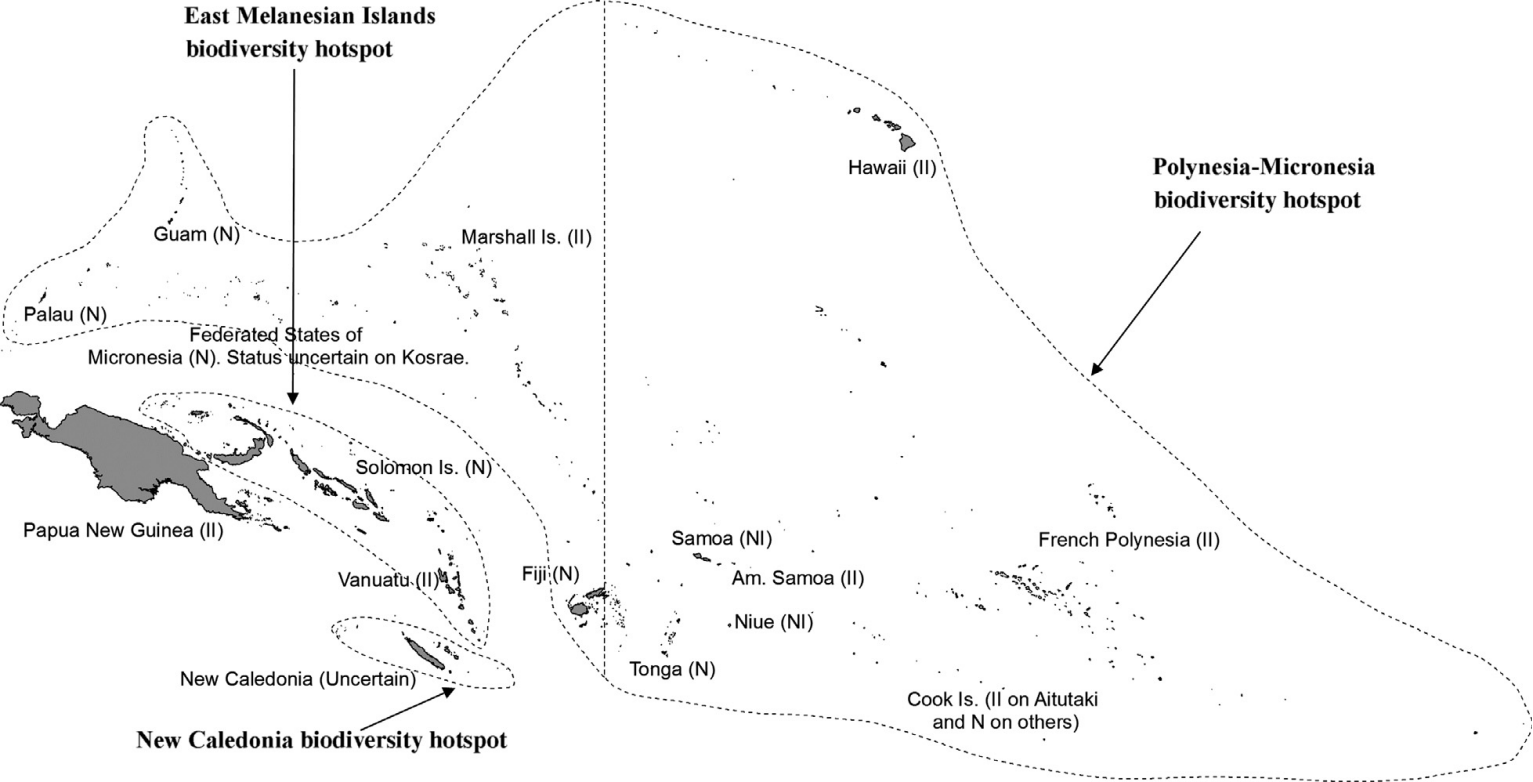
Pacific island countries where *M. peltata* is present and is native (N), native invasive (NI) and introduced invasive (II). Status data are adapted from PIER (2009). Broken lines show the extent of the three biodiversity hotspots in the region.

*Merremia peltata* (L.) Merrill (Convolvulaceae) is a woody vine with a geographical range extending from East Africa to Tahiti (Whistler 1995; Master et al. 2013). It is considered native to Madagascar, Mauritius, La Réunion, Tanzania (Pemba Island), Indonesia, Malaysia, the Philippines and Australia (northern Queensland) (Paynter et al. 2006). The islands of the Pacific region have been included within its native area; however, its status in this region is uncertain (Meyer 2000, Space and Flynn 2002; Whistler 2002; PIER, 2009) (Fig. 1). In the Pacific, it has been suggested as a native to some countries such as Samoa, Cook Islands, Federated States of Micronesia (FSM), Fiji, French Polynesia, the Solomon Islands and Niue (PIER, 2009). On the other hand, it has been identified as an invasive species of environmental concern in several other Pacific island countries such as some islands of American Samoa, Marshall Islands, Papua New Guinea (PNG) and Vanuatu (Meyer 2000; Space and Flynn 2002; Whistler 2002; PIER, 2009) while its status is uncertain in New Caledonia (PIER, 2009) (Fig. 2). The resolution of this uncertain status requires an understanding of the biogeography of *M. peltata* and genetic studies using molecular markers are useful for this (Palmer et al. 2010). This genetic information should underpin management options for this species in the Pacific region.

*M. peltata* is a common species found in dry lowland and mesic inland vegetation types in the Pacific region (Meyer 2000); however, in Samoa, it has been noted as a dominant species on the landscape only during the past two decades (Kirkham 2005). It reproduces both vegetatively and by seed; however, the seed viability rates are reported to be low (Bacon 1982). Viable seeds can be transported by sea to new islands (Hacker 1990) but the distance and duration that seeds can travel is largely unknown. Dispersal by sea may have led to its presence on the remote islands of the Pacific region. It is not known whether animals play a role in pollination and dispersal of this species as according to Kirkham (2005), in Samoa, “ants were observed in the corolla of the *M. peltata* flowers, but never bees or wasps that were frequently seen on flowers of other species”.

In parts of the Pacific region, *M. peltata* is considered a troublesome weed, capable of smothering trees up to 20 m tall (GBIF, 2010). It commonly occurs in plantations and forest clearings where it often suppresses other important vegetation and regeneration of native species (Bacon 1982; Bakeo and Qarani 2005; Kiapranis and Nimiago 2005; Wood 2012). Many local and academic experts consider it to be a serious threat to the native ecosystems of the Pacific islands (Meyer 2000). Furthermore, a weed risk assessment undertaken for this species for Australia and Hawaii resulted in a rating of “high risk” indicating that *M. peltata* posed a high risk of becoming a serious pest (PIER, 2009). It has also been suggested as a serious weed for which biological control should be explored in this region (Dovey et al. 2004). However, any uncertainty associated with the status of a species needs to be resolved through investigations into its genetics (Dodd and Hayes 2009, 2010; Paynter et al. 2009). Biological control should not be suggested for management of any species within its native range because their regulating natural enemies are already present and they may have expanded their range due to other factors such as overgrazing (Pemberton 1984).

Some researchers argue that *M. peltata* is native to the region (Space and Flynn 2002; Whistler 2002). In countries such as Samoa, frequent disturbance caused by hurricanes or shifting cultivation can increase its distribution. In Samoa, it also appeared to play an important role in rainforest regeneration and attempts to control it could promote other invasive species (Kirkham 2005). Kirkham (2005) found that *M. peltata* behaved differently depending on whether it occurred in the forest canopy or as ground cover. It was found to suppress species diversity when acting as ground cover and also aided the spread of other invasive vines such as *Mikania micrantha*; however, it seemed to support species diversity when occurring in the forest canopy. The dominance of *M. peltata* is driven by fluctuating patterns of disturbance on the landscape, both anthropogenic and natural. The anthropogenic disturbance has been mainly caused by changes in land‐use due to increase in agriculture, while disturbances caused by tropical cyclones also have an effect on the dominance of *M. peltata* (Kirkham 2005).

The lack of agreement regarding the status of *M. peltata* throughout the region is problematic in terms of control strategies (PIER, 2009; Dodd and Hayes 2010). Manual control is difficult given its predominantly vegetative mode of reproduction since root fragments re‐sprout and stem fragments readily take root (Paynter et al. 2006). Chemical control using glyphosate has proved to be effective against *M. peltata* in forestry plantations in some countries like the Solomon Islands (Miller 1982). Biological control has been proposed although the controversial issue of its native/invasive status in the Pacific region has complicated matters (Dovey et al. 2004; Dodd and Hayes 2010). Native weed species have an important ecological role and reduction of these species could have a destabilizing impact on ecosystems. Thus, in the case of *M. peltata* it would be prudent to undertake further research to ascertain its status to assess the suitability of biological control. It is also important that any control strategies for *M. peltata* are underpinned by information describing its potential distribution. Climate change will add to the complications of managing such a species because changes in climate may lead to shifts in the climatic limits that usually constrain its range (Taylor et al. 2012a). An estimate of how the potential range of *M. peltata* is likely to shift under climate change would contribute to an understanding of the potential impact of the species in the Pacific region.

To this end, CLIMEX, a popular species distribution modeling software for undertaking risk assessments for weeds (Chejara et al. 2010; Taylor and Kumar 2013a,b), was used to develop a model of the climate responses of *M. peltata*. A range of information, such as experimental observations of its growth response to temperature and soil moisture, current distribution, and seasonal phenology, were used in model development. This model was then used to examine its potential current distribution and future distribution under climate change on some of the main archipelagos in the South Pacific. Despite rapid advances in the development of gridded data sets for global climate normals (Hijmans et al. 2005; Mitchell and Jones 2005), these datasets are still inappropriate for very small islands because they are unlikely to reflect the variations in climate that can occur over small distances on mountainous islands (Taylor and Kumar 2013a). Hence, results are only presented for the following archipelagos: Fiji, Hawaii, New Caledonia, Papua New Guinea, Solomon Islands and Vanuatu.

## Materials and Methods

### CLIMEX software

CLIMEX for Windows Version 3 has been widely used to model the potential distribution of a range of species (Sutherst and Maywald 1985; Chejara et al. 2010; Webber et al. 2011; Taylor et al. 2012b). This software was used in the present study to investigate the impacts of climate change on the potential distribution of *Merremia peltata*. CLIMEX is based primarily on the response of a species to climate and does not include other factors such as biotic interactions that may affect its distribution (Sutherst and Maywald 1985). The following references provide a full description of the theory behind the development of the CLIMEX software and its applications in modeling potential species' distributions (Sutherst and Maywald 1985; Sutherst 2003; Sutherst et al. 2007). The final output of the CLIMEX model is the Ecoclimatic index (EI) value which indicates the climatic suitability of a location for the persistence of the species under investigation. The EI values range from 0 to 100; a location with an EI value of 0 is deemed unsuitable, 1–10 indicate marginal habitats, 10–20 indicate suitable habitats and values >20 are highly suitable (Sutherst and Maywald 1985; Taylor et al. 2012b).

### Climate data and climate change scenarios

The CliMond website provides historical climatic data for the period 1961–1990 as well as future climate for 2030 and 2100 at 10' resolution (Kriticos et al. 2011). Five climatic variables were utilized in this study; average minimum monthly temperature (*T*_min_), average maximum monthly temperature (*T*_max_), average monthly precipitation (*P*_total_) and relative humidity at 09:00 h (RH_09:00_) and 15:00 h (RH_15:00_) (Kriticos et al. 2011; Taylor et al. 2012b). Two Global Climate Models (GCMs), CSIRO‐Mk3.0 (CS) (Gordon et al. 2002) and MIROC‐H (MR) (Centre for Climate Research, Japan) and the A1B and A2 SRES scenarios (IPCC, 2007) were used in this study (Taylor et al. 2012b). The following published research provides a full explanation of GCM and scenario selection (Hennessy and Colman 2007; Rahmstorf et al. 2007; Kriticos et al. 2011; Taylor et al. 2012b).

### Present distribution of *M. peltata*

Information on the global distribution of *M. peltata* was downloaded from the Global Biodiversity Information Facility (GBIF, 2010), the Pacific Island Ecosystems at Risk (PIER, 2009), Australia's Virtual Herbarium (Australia's Virtual Herbarium 2009) databases and various sources of literature (Smith 1991; Josekutty et al. 2002; Kirkham 2004, 2005; Paynter et al. 2006; Master et al. 2013). A total of 236 records were collected of which only 173 were used after checking and removing duplicate points (locations with the same latitude and longitude value) and records with no geographic coordinates. This is a necessary part of data quality control since only verified location points with a latitude and longitude value can be used to inform the parameter fitting process. The distribution records from Australian were set aside for validation and not used in the parameter fitting process. This ensured that a set of independent observations of naturalized populations from Australia was available for model validation.

### Fitting CLIMEX parameters

Table 1 summarizes the parameters used in the CLIMEX model for *M. peltata*. For a full description of the model parameters see Sutherst and Maywald (1985). A manual iterative procedure is used in CLIMEX to fit parameters. During this procedure, parameters are adjusted and the model is run (Kriticos et al. 2005). A visual comparison between the various indices and the species distribution data is undertaken. This procedure is repeated until a satisfactory level of agreement between the model results and the distribution data is obtained with higher EI values coinciding with areas of recorded distribution (Shabani et al. 2012). The derivation of the parameter values are discussed below. CLIMEX utilizes meteorological data that represents long‐term monthly averages, not daily values. Thus, it is not possible to make direct comparisons between parameter values that were derived using the model and directly observed instantaneous values (Kriticos et al. 2003).

**Table 1:** CLIMEX parameter values used for *M. peltata*

Index	Parameter	Value
Temperature	DVO = lower threshold	15°C
	DV1 = lower optimum temperature	18°C
	DV2 = upper optimum temperature	30°C
	DV3 = upper threshold	33°C
	PDD = degree‐day threshold (minimum annual total number of degree‐days above 15°C (DV0) needed for population persistence	2900°C days
Moisture	SM0 = lower soil moisture threshold	0.35
	SM1 = lower optimum soil moisture	0.6
	SM2 = upper optimum soil moisture	1.3
	SM3 = upper soil moisture threshold	2
Cold stress	TTCS = temperature threshold	10°C
	THCS = stress accumulation rate	−0.003 week^−1^
	DTCS = Minimum degree‐day cold stress threshold	20°C days
	DHCS = Degree‐day cold stress rate	−0.003 week^−1^
Heat stress	TTHS = temperature threshold	33°C
	THHS = stress accumulation rate	0.002 week^−1^
Dry stress	SMDS = threshold soil moisture	0.35
	HDS = stress accumulation rate	−0.001 week^−1^
Wet Stress	SMWS = threshold soil moisture	2
	HWS = stress accumulation rate	0.002 week^−1^

Stress parameters were fitted using the known native distribution in Africa and tropical Asia and the naturalized distribution in the South Pacific (PIER, 2009). Growth parameters were fitted using information on the ecophysiology of *M. peltata* (Bacon 1982; Smith 1991; Josekutty et al. 2002; Kirkham 2004, 2005; Paynter et al. 2006; Master et al. 2013). The Compare Locations model in CLIMEX was used with the Wet Tropical template which most closely reflected the climatic requirements of *M. peltata*. The parameters were iteratively adjusted as described above. The parameters were checked to ensure that they were biologically reasonable (Taylor et al. 2012b).

### Cold stress

The global occurrences of *M. peltata* have been reported between 27°N and 25°S (Australia's Virtual Herbarium 2009, PIER, 2009, GBIF, 2010). Consequently, the southern and northern limits of *M. peltata's* global distribution were defined using two cold stress mechanisms. The cold stress temperature threshold (TTCS) was set at 10°C with the stress accumulation rate (THCS) set at −0.003 week^−1^ while the Cold‐Stress Degree‐day Threshold (DTCS) was set at 20°C days, with the stress accumulation rate (DHCS) set at −0.003 week^−1^. These two mechanisms ensured that the potential distribution was restricted to the known northern and southern limits (Taylor et al. 2012b).

### Heat stress

The heat stress parameter (TTHS) was set at 33°C, the same level as the limiting high temperature (DV3) with a stress accumulation rate (THHS) of 0.002 week^−1^.

### Dry stress

The dry stress parameter was set at the same level (0.35) as the lower soil moisture threshold (SM0) because soil moisture related stresses probably begin at the same soil moisture levels where growth stops (Kriticos et al. 2003). The stress accumulation rate (HDS) was set at −0.001 week^−1^.

### Wet stress

The wet stress threshold (SMWS) was set to 2 and the stress accumulation rate (HWS) was set at 0.002 week^−1^ since the ideal rainfall requirements of *M. peltata* exceed 2400 mm annually (Paynter et al. 2006).

### Temperature index

*M. peltata* distribution is restricted to warm tropical regions (Paynter et al. 2006). Using the Wet Tropical species template as a starting point, the minimum (DV0) and maximum (DV3) threshold temperatures were set at 15 and 33°C, respectively. The lower (DV1) and upper (DV2) optimal temperatures were set at 18 and 30°C, respectively, based on the response of similar tropical vines. These provided a good fit to the observed global distribution (Taylor et al. 2012b).

### Moisture index

*M. peltata* requires rainfall levels of above 2400 mm and it will tolerate rainfall levels of up to 3200 mm per year (Paynter et al. 2006). Thus, the lower moisture threshold (SM0) was set at 0.35, the lower (SM1) and upper (SM2) optimal soil moisture were set at 0.6 and 1.3, respectively, and the limiting soil moisture (SM3) was set at 2.

### Degree day threshold

The minimum amount of thermal accumulation necessary to complete one generation depends on the length of the growing season which is calculated from the PDD and DV0 parameters. PDD is the number of degree‐days of thermal accumulation above DV0 required by a species to complete one generation (Kriticos et al. 2003). The plant cannot reproduce if this amount of heat is not available. A threshold parameter for PDD of 2900 degree‐days above DV0 (15°C) was fitted to the native African distribution as this value just allowed the persistence of this species at its limits of distribution in Southern Africa.

## Results

### Model validation and historical climate

**Figure 3:**
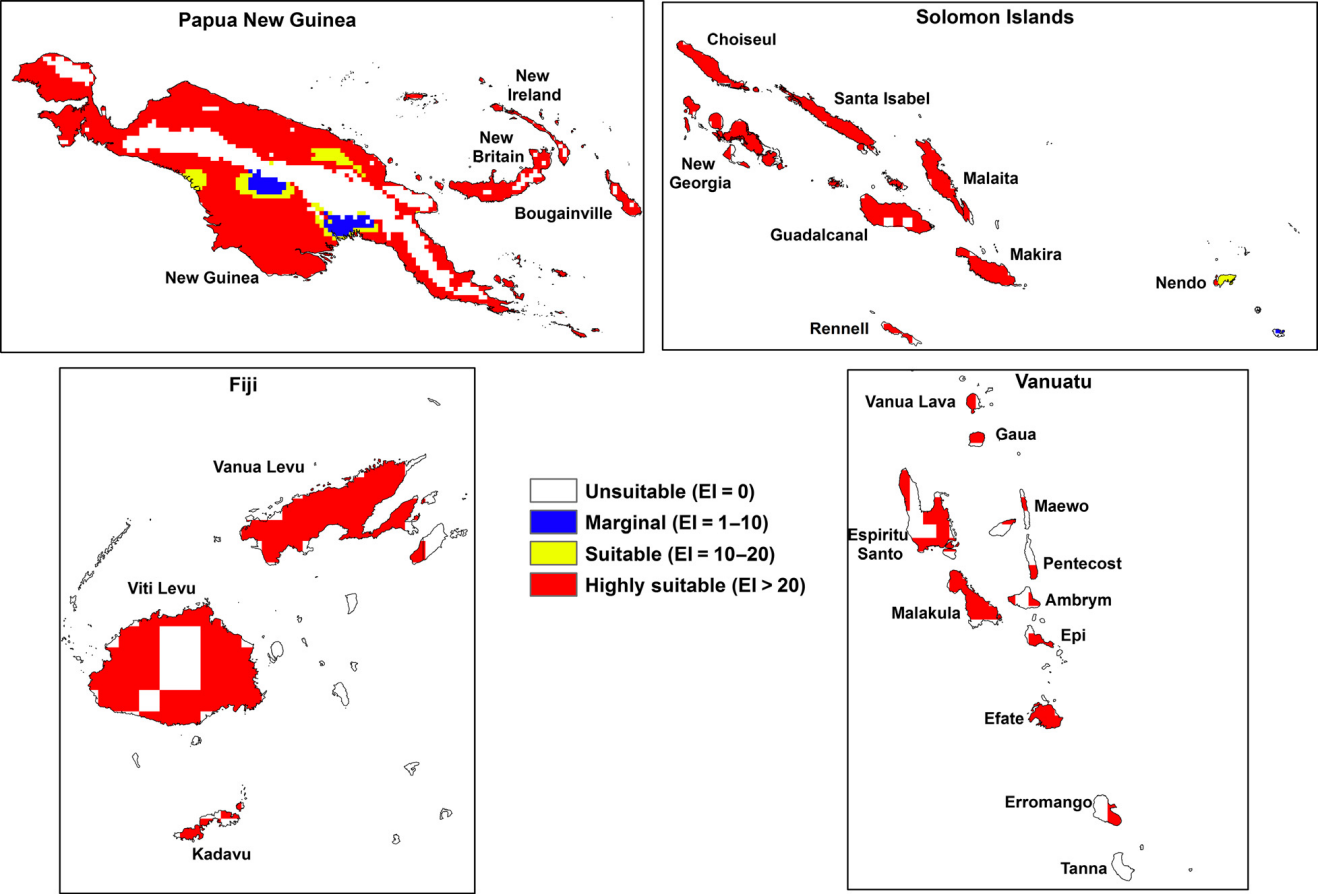
The archipelagos with large areas of suitable to highly suitable climate (EI) for *M. peltata* under reference climate (averaging period 1950–2000).

The results presented here only focus on the following archipelagos: Fiji, Hawaii, New Caledonia, Papua New Guinea, Solomon Islands and Vanuatu. Under historical climate, areas of climatic suitability for *M. peltata* were identified in Fiji, Papua New Guinea, Solomon Islands and Vanuatu (Fig. 3). Changes in the area of climatic suitability for *M. peltata* are shown in Table 2.

**Table 2:** Areas of *Merremia peltata* suitability under different scenarios for the Pacific region

Scenario	Area (km^2^)			
	Unsuitable	Marginal	Suitable	Highly suitable
Current	248508	24624	38880	625644
2030 A1B CS	208008	55404	117936	556308
2030 A2 CS	210600	50220	106596	570240
2030 A1B MR	209628	58644	104004	565380
2030 A2 MR	209952	55404	96552	575748
2100 A1B CS	448092	177552	81648	230364
2100 A2 CS	567324	133164	61560	175608
2100 A1B MR	483732	126684	67716	259524
2100 A2 MR	586764	98172	56700	196020

**Figure 4:**
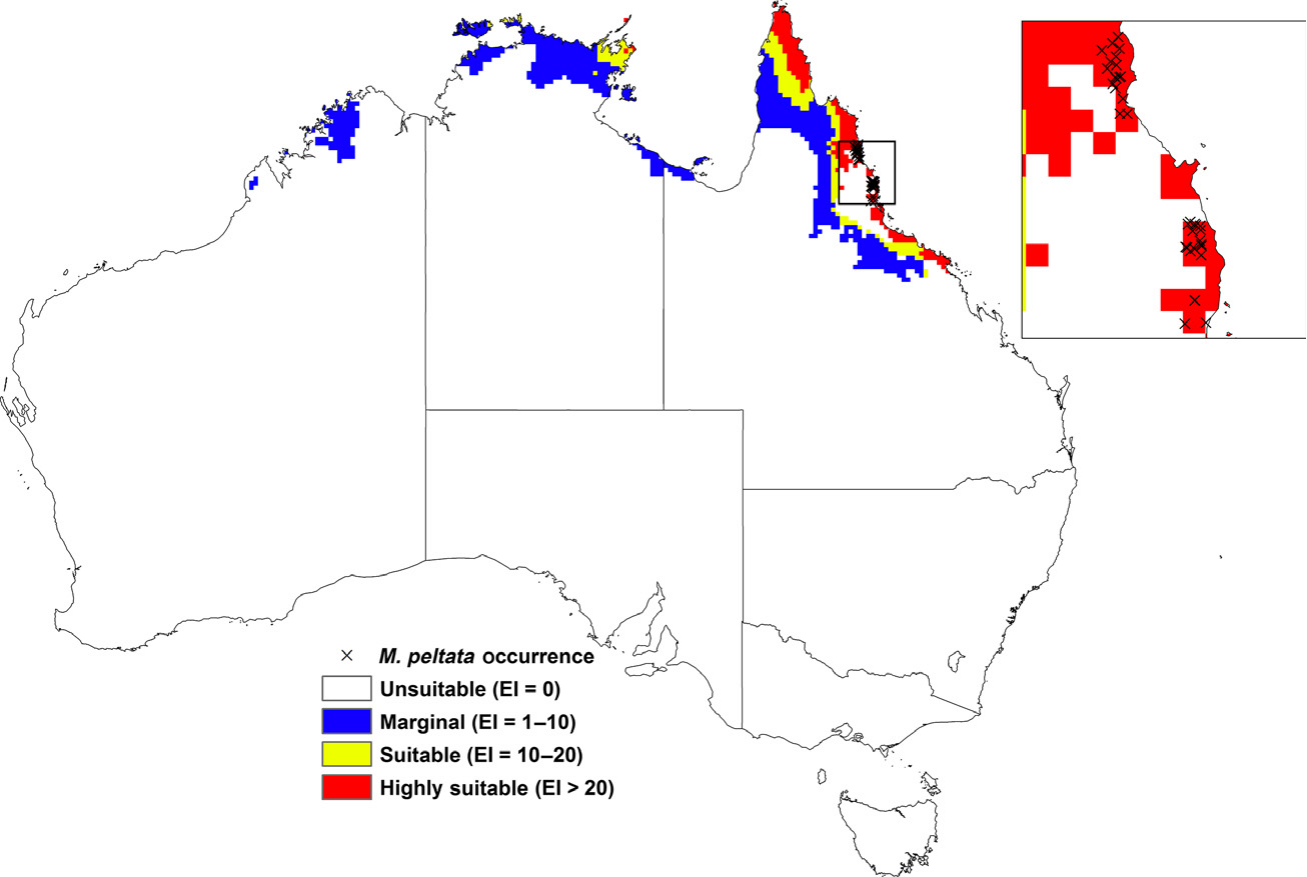
Current and modeled climate (EI) for *M. peltata* based on CLIMEX for reference climate (averaging period 1950–2000). Data for current Australian distribution is taken from Australia's Virtual Herbarium.

The potential distribution of *M. peltata* together with its occurrence in Australia is shown in Figure 4. Approximately 77% of the occurrence records fall within the highly suitable category. In Australia, the model projects coastal areas of Northern Queensland from Cape York to Bowen as well as small parts of the coastal region of Arnhem Land in the Northern Territory to have suitable to highly suitable climate for *M. peltata* (Fig. 4). Central Australia and other parts of the continent are projected to be climatically unsuitable for this species mainly due to dry stress based on its high rainfall requirements (Paynter et al. 2006).

### Future climate

**Figure 5:**
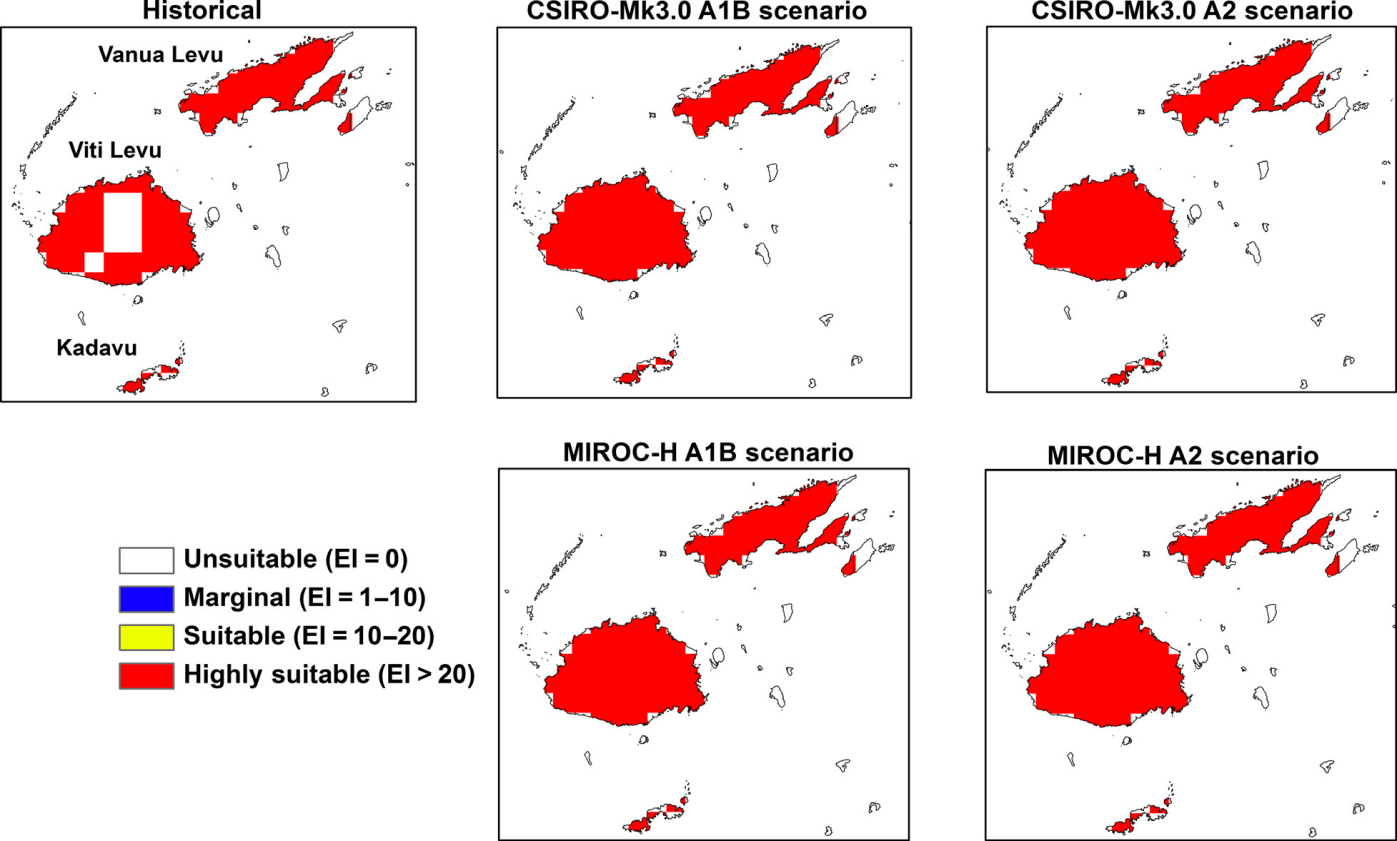
The climate (EI) for *M. peltata* in Fiji for 2100.

**Figure 6:**
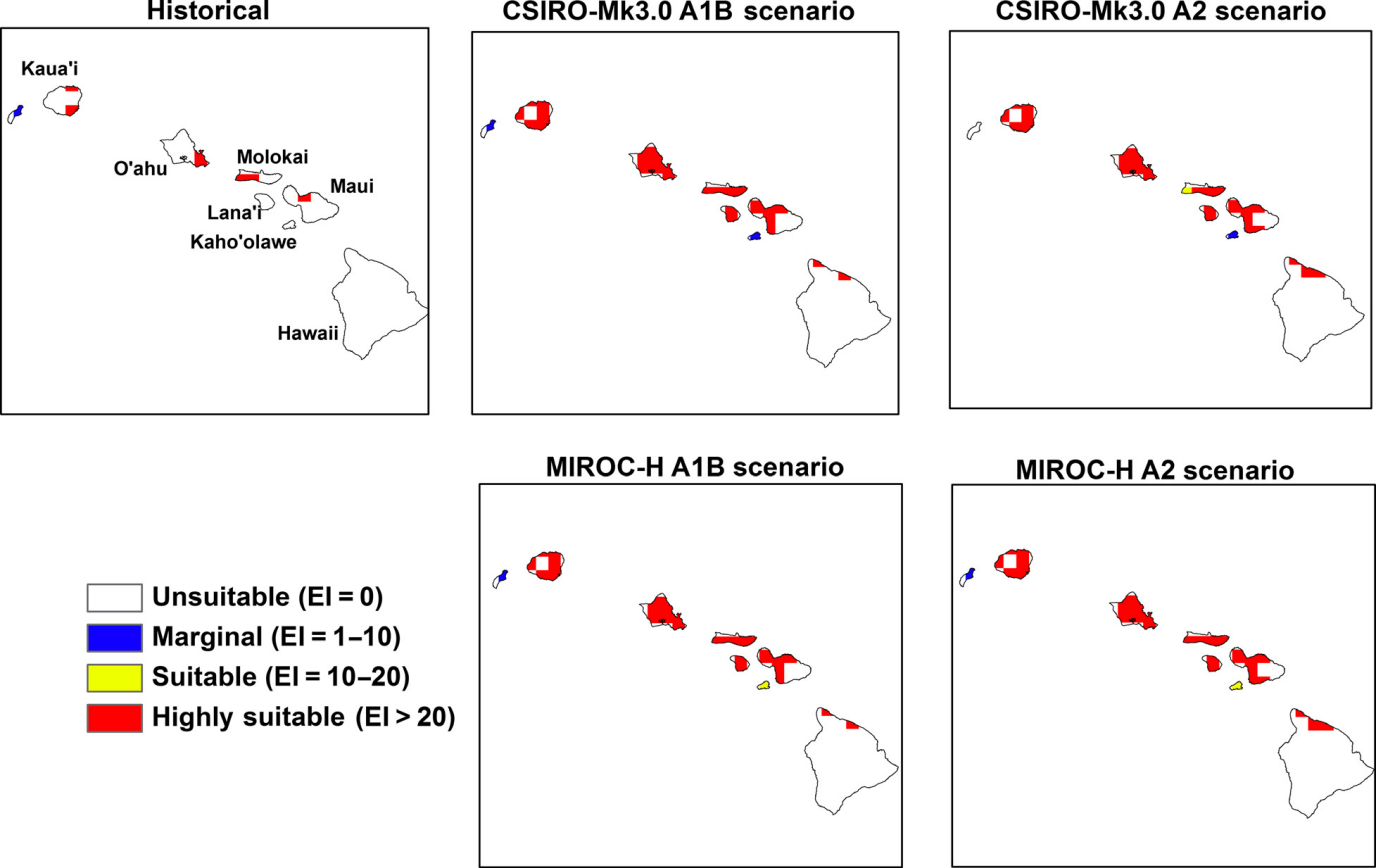
The climate (EI) for *M. peltata* in Hawaii for 2100.

**Figure 7:**
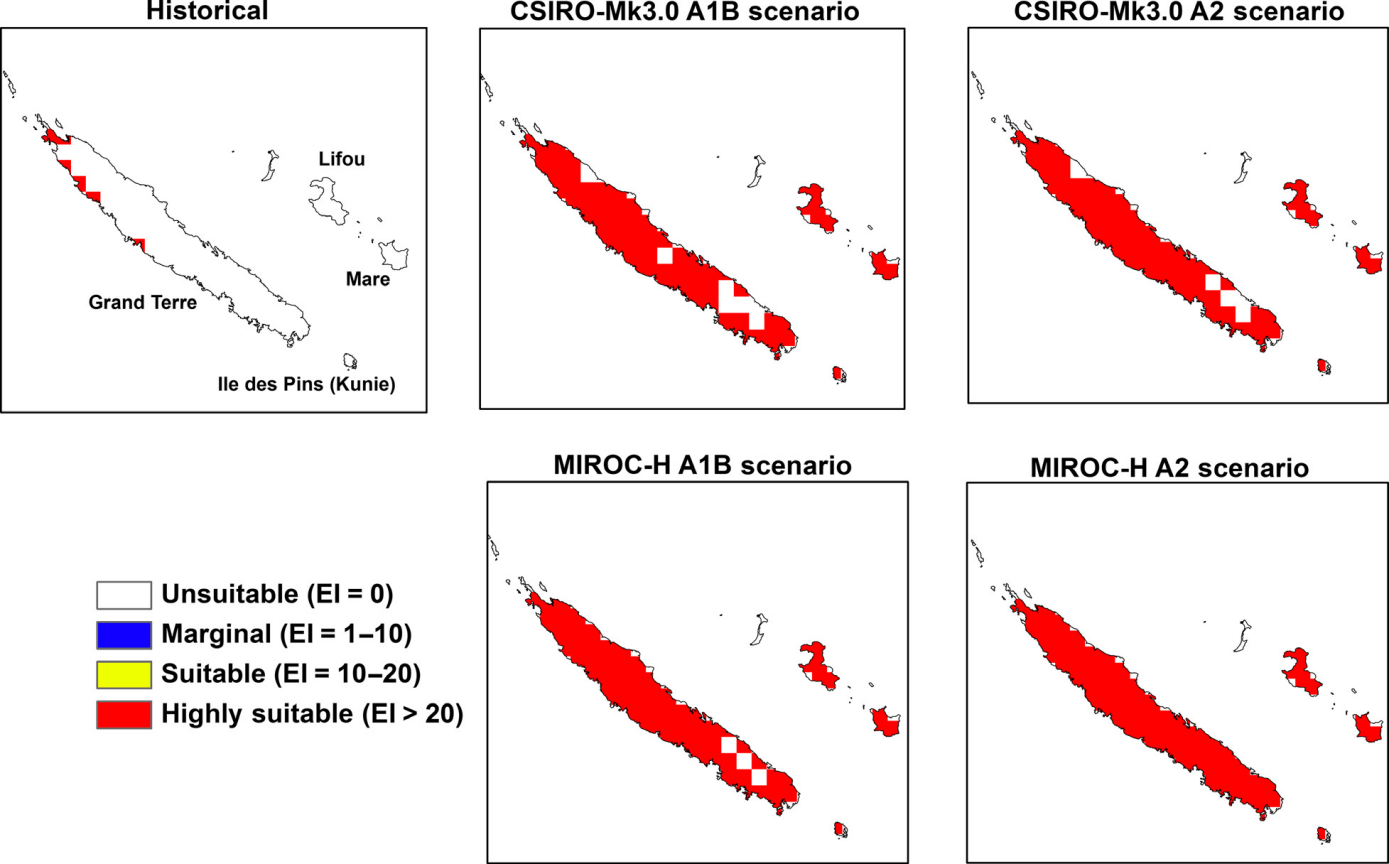
The climate (EI) for *M. peltata* in New Caledonia for 2100.

**Figure 8:**
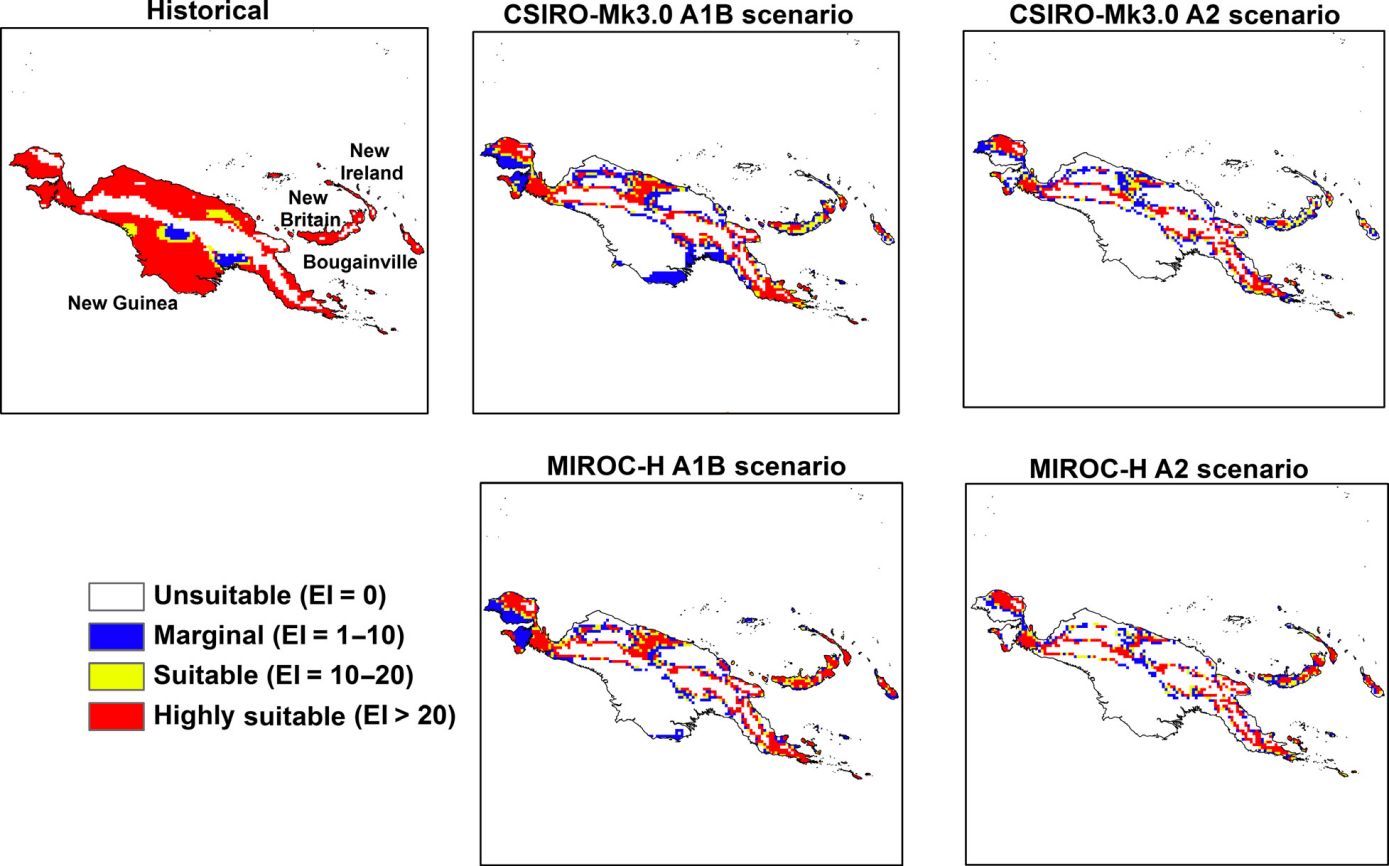
The climate (EI) for *M. peltata* in Papua New Guinea for 2100.

**Figure 9:**
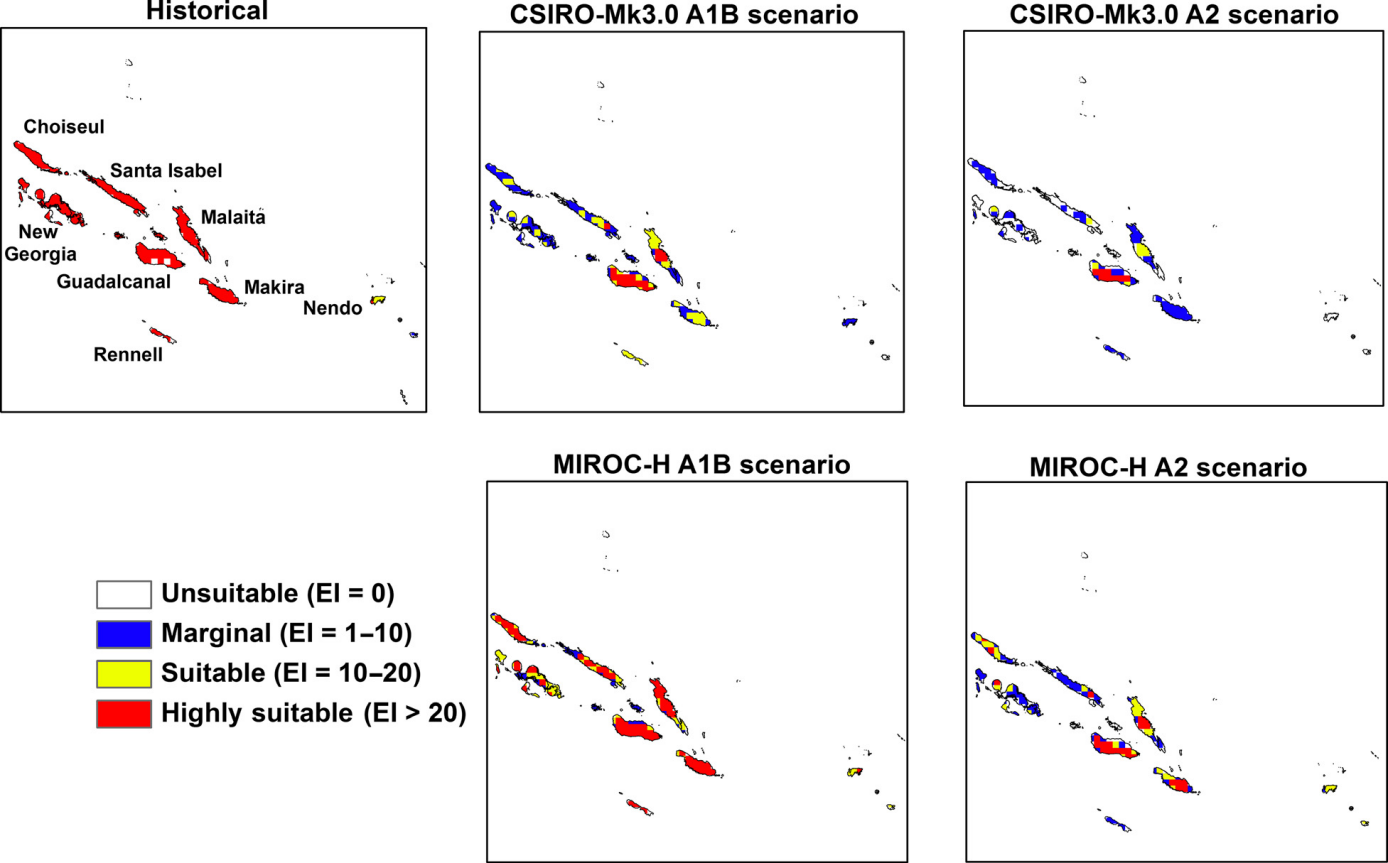
The climate (EI) for *M. peltata* in Solomon Islands for 2100.

**Figure 10:**
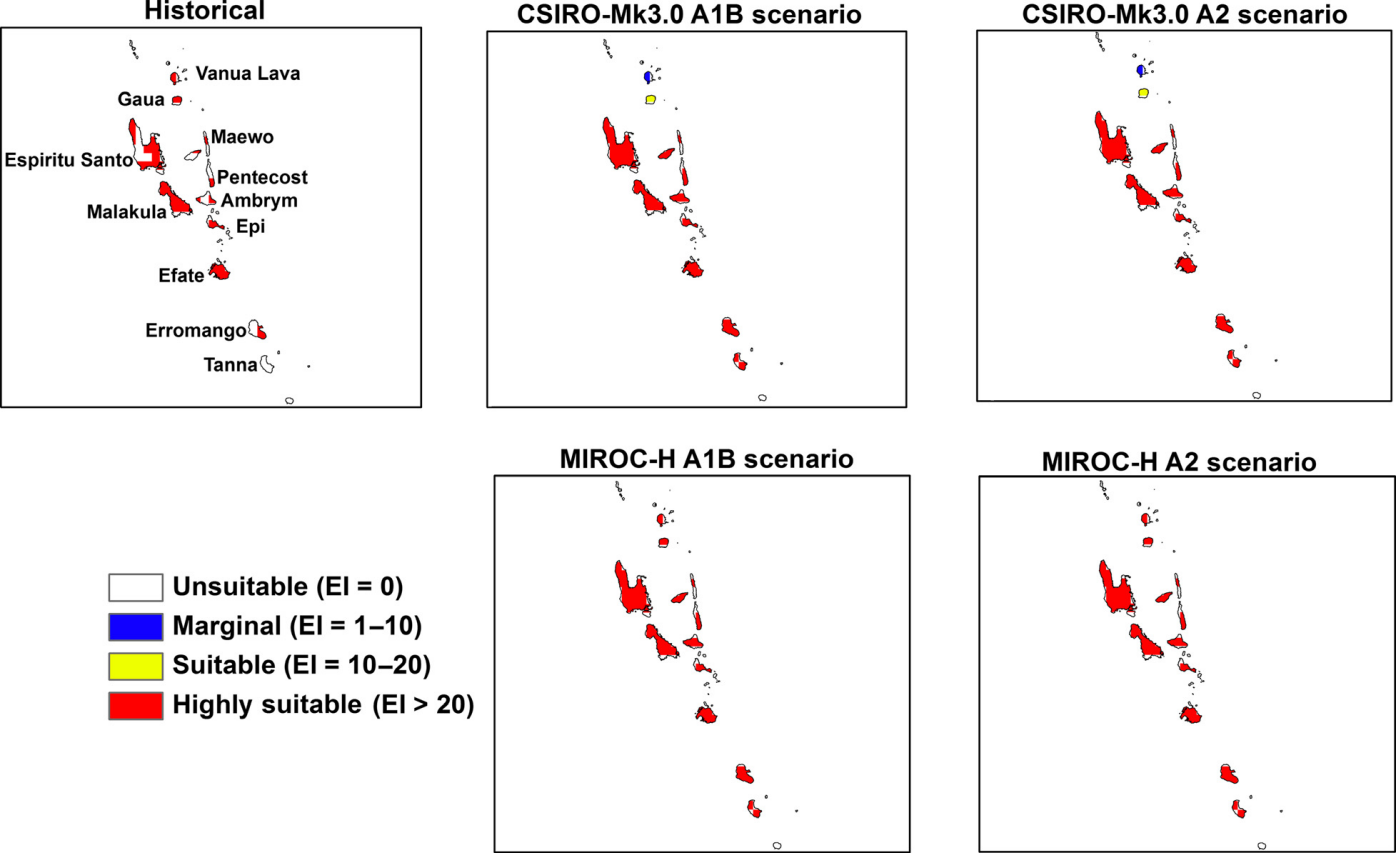
The climate (EI) for *M. peltata* in Vanuatu for 2100.

Large areas in the region will become unsuitable or marginal for *M. peltata* with a further change in climatic suitability from highly suitable to merely suitable by the end of the century (Table 2). The locations of these changes in climatic suitability by the end of the century are shown in Figures 5, 6, 7, 8, 9, 10. Results for 2030 are shown in supplementary materials S1–S6. The general trends indicate range expansions in Fiji, Hawaii, New Caledonia and Vanuatu for this species while range contractions are indicated for PNG and Solomon Islands by the end of the century. The major change observed in the potential distribution of *M. peltata* in Fiji occurs in the central region of Viti Levu. Under current climate, this region is projected as climatically unsuitable but becomes highly suitable under future climate. Furthermore, the climate on the islands of Vanua Levu and Kadavu remain highly suitable for *M. peltata* until 2100 (Fig. 5). In Hawaii, range expansions are seen in Kaua'i, O'ahu, Molokai, Maui and the northern coast of the main island of Hawaii (Fig. 6). Additionally, the two smaller islands of Lana'i and Kaho'olawe, which are unsuitable for *M. peltata* under current climate, become suitable under future climate scenarios, particularly with the MIROC‐H GCM.

Substantial range expansions are indicated for the main island of Grand Terre in New Caledonia. Other islands which are projected as unsuitable under current climate, such as Ile des Pins (Kunie), Mare and Lifou, become highly suitable under future climate (Fig. 7).

Papua New Guinea remains climatically suitable for *M. peltata* until 2030 (S4); however, substantial contractions in suitable range occur by the end of the century on all the large islands in this archipelago. This range contraction is more pronounced under the A2 scenario (Fig. 8).

Islands in the Solomon Islands archipelago remain climatically suitable for *M. peltata* until 2030 (S5). By the end of the century, however, climatic suitability for this species diminishes on all the islands, especially under the A2 scenario. The islands of Guadalcanal, Makira, Rennell and Malaita remain highly suitable until 2100 under the A1B scenario with the MIROC‐H GCM (Fig. 9). In Vanuatu, range expansions are indicated for islands which are unsuitable under current climate, such as Erromango and Tanna. Further increases in suitable range for *M. peltata* can be seen on Espiritu Santo where larger sections of the island become highly suitable compared to current climate (Fig. 10).

## Discussion

The potential distribution of *M. peltata* was modeled for historical climate and under future climate scenarios. The variable impacts that climate change may have on this species' potential distribution in the Pacific region were highlighted. The model provided a good fit to the current distribution of this species in the region as well as the Australian distribution which was reserved for model validation purposes. In general, range expansions are indicated for Fiji, Hawaii, New Caledonia and Vanuatu while a reduction in climatically suitable areas is indicated for PNG and Solomon Islands under future climate scenarios.

The two GCMs are consistent in their projections of changes in potential distribution of *M. peltata* under future climate for Fiji, Hawaii and Vanuatu while some differences can be seen in the projections for the other four countries. In general the MIROC‐H projections indicate that larger areas will remain climatically suitable for *M. peltata* in the future compared to CSIRO‐Mk3.0. The MIROC‐H GCM predicts a temperature increase of approximately 4.31°C, while the CSIRO‐Mk3.0 GCM predicts an increase of 2.11°C by 2100 (Kriticos et al. 2011). Their predictions for changes in precipitation levels also differ with CSIRO‐Mk3.0 predicting a 14% decrease in future mean annual rainfall while MIROC‐H predicts a 1% decrease (Chiew et al. 2009). *M. peltata* is restricted by low rainfall (Paynter et al. 2006) and so the larger decreases in rainfall predicted by CSIRO‐Mk3.0 would lead to reduced climatic suitability in the future under this GCM.

Some archipelagos within the introduced and invasive range of *M. peltata* were identified as remaining climatically suitable for this species well into the future with range expansions projected for some of the small islands in these archipelagos that currently do not have *M. peltata*. In these cases strategic control measures will be required to prevent its spread to presently unoccupied islands. The isolation and smaller size of these islands place them in a better position to prevent entry into areas that are currently unsuitable for *M. peltata* but will become climatically suitable in the future. However, for such measures to be successful, quarantine regulations and their enforcement will need to be strengthened. Furthermore, simple and low‐cost strategies such as weed alerts and low‐cost surveillance, especially around airports and ports, may be a worthwhile investment on the part of biosecurity agencies in these archipelagos (Taylor et al. 2012b). The decrease in climatic suitability in the introduced and invasive range of *M. peltata* by the end of the century is encouraging, although many islands in these areas will likely remain climatically suitable in the short term. These results should be useful in prioritizing areas for eradication and in determining areas where containment would be cost‐effective (Taylor and Kumar 2013a).

Future range expansions within the native range of *M. peltata* are positive; however, range contractions are of concern. The distribution maps from this study could be used to make decisions about conservation implications, particularly on islands where climate is projected to become unsuitable for this species by 2100. The projected distribution maps identify some areas within its native range where this species can persist as anthropogenic climate change progresses. In such cases, focusing management efforts on the changing disturbance patterns, both natural and anthropogenic may be useful. The investigation into the response of species such as *M. peltata* to changes in climate serves a dual purpose. The results can be used to inform biosecurity planning by assessing threat levels to native biodiversity on islands where it is introduced and invasive. They can also inform management and conservation strategies on islands where it is native but has become dominant due to other factors such as disturbance.

All species may respond in unpredictable ways to multiple interacting factors associated with global change and this also applies to species that behave invasively (Bradley et al. 2010). Rising temperatures and altered precipitation together with increased availability of resources from higher carbon dioxide levels and nitrogen deposition, as well as land use change will impact invasive species distributions (Dukes 2000; Hulme 2009). However, these impacts will not be limited to single species in isolation but will affect whole ecosystems. Therefore, the issue of future changes to the distribution of *M. peltata* will need to be viewed in this context of wider changes. Integrated assessments that investigate large‐scale changes to entire ecosystems in the Pacific region will be beneficial for managers coping with the impacts of global change on biodiversity. However, this can be a demanding task, both in terms of funding and organization due to the comprehensive and interdisciplinary nature of such assessments (Bradley et al. 2010). The findings from species‐specific studies such as the one reported here can be useful for managing the impacts of climate change on biodiversity in the short term but also potentially contribute to broad scale assessments in the long‐term.

### Model limitations and future research

CLIMEX is based on the response of a species to climate and does not explicitly include nonclimatic factors such as dispersal potential and biotic interactions in the modeling process Taylor et al. 2012b). The uncertainty related to future global greenhouse gas emission patterns also introduce further uncertainty to model projections (Kriticos et al. 2006). Therefore, the results presented here should be interpreted as providing an *indication* of the direction and magnitude of change that may be expected in the future (Taylor et al. 2012b). The distribution maps provided here show areas of climatic suitability for *M. peltata* and should not be treated as predicted future distributions. Other factors like lack of dispersal opportunities will also play a role in this species' potential future distribution.

The gridded climate datasets used in this study have a coarse resolution and may not reflect the climatic variation that can occur over small distances on the mountainous islands in the Pacific region. However, in the absence of finer resolution data sets, this study has utilized the best available data to perform the climate change modeling.

## Conflict of Interest

None declared.

## Supporting information


**Figure S1.** The climate (EI) for *M. peltata* in Fiji for 2030.

**Figure S2.** The climate (EI) for *M. peltata* in Hawaii for 2030.

**Figure S3.** The climate (EI) for *M. peltata* in New Caledonia for 2030.

**Figure S4.** The climate (EI) for *M. peltata* in Papua New Guinea for 2030.

**Figure S5.** The climate (EI) for *M. peltata* in Solomon Islands for 2030.

**Figure S6.** The climate (EI) for *M. peltata* in Vanuatu for 2030.
