# Supplementary figures and images for: Will climate change impact the potential distribution of a native vine (Merremia peltata) which is behaving invasively in the Pacific region?
Source: Ecol Evol. 2016 Jan 11;6(3):742–54. doi: 10.1002/ece3.1915 (PMC4739570; doi:10.1002/ece3.1915)

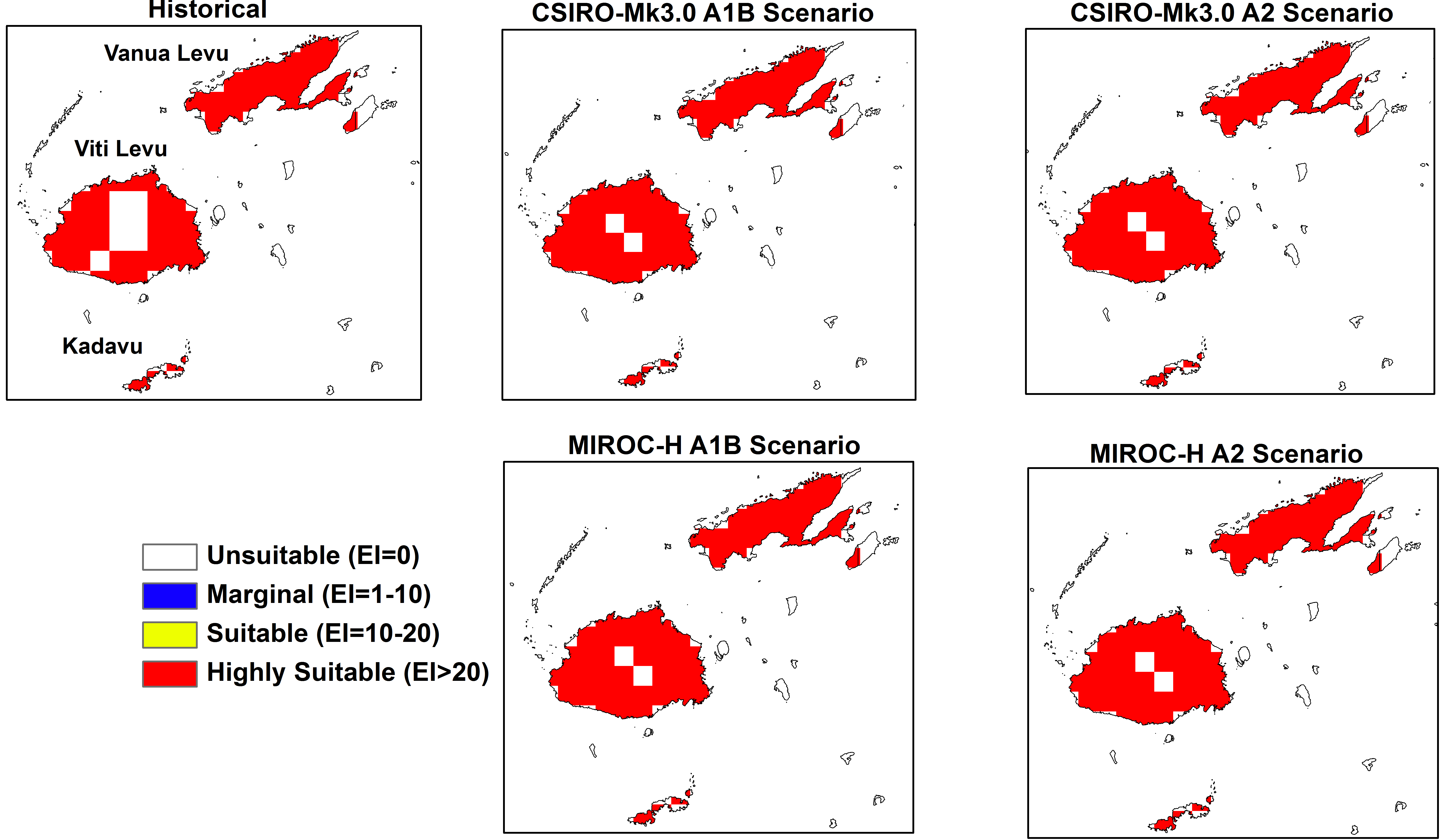

Supplement: Supplementary file 1 — Figure S1. The climate (EI) for M. peltata in Fiji for 2030. [file ECE3-6-742-s001.tif]

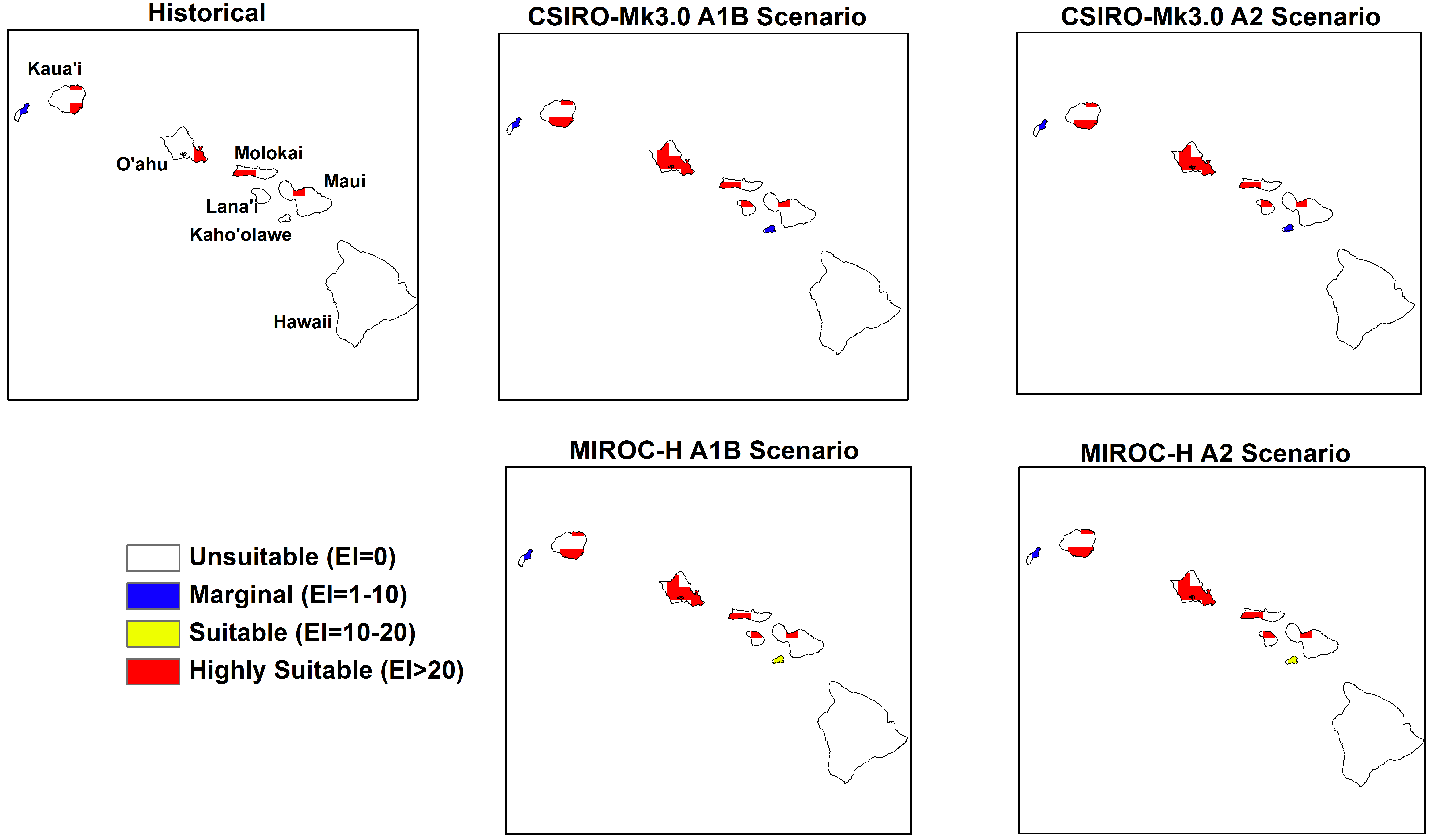

Supplement: Supplementary file 2 — Figure S2. The climate (EI) for M. peltata in Hawaii for 2030. [file ECE3-6-742-s002.tif]

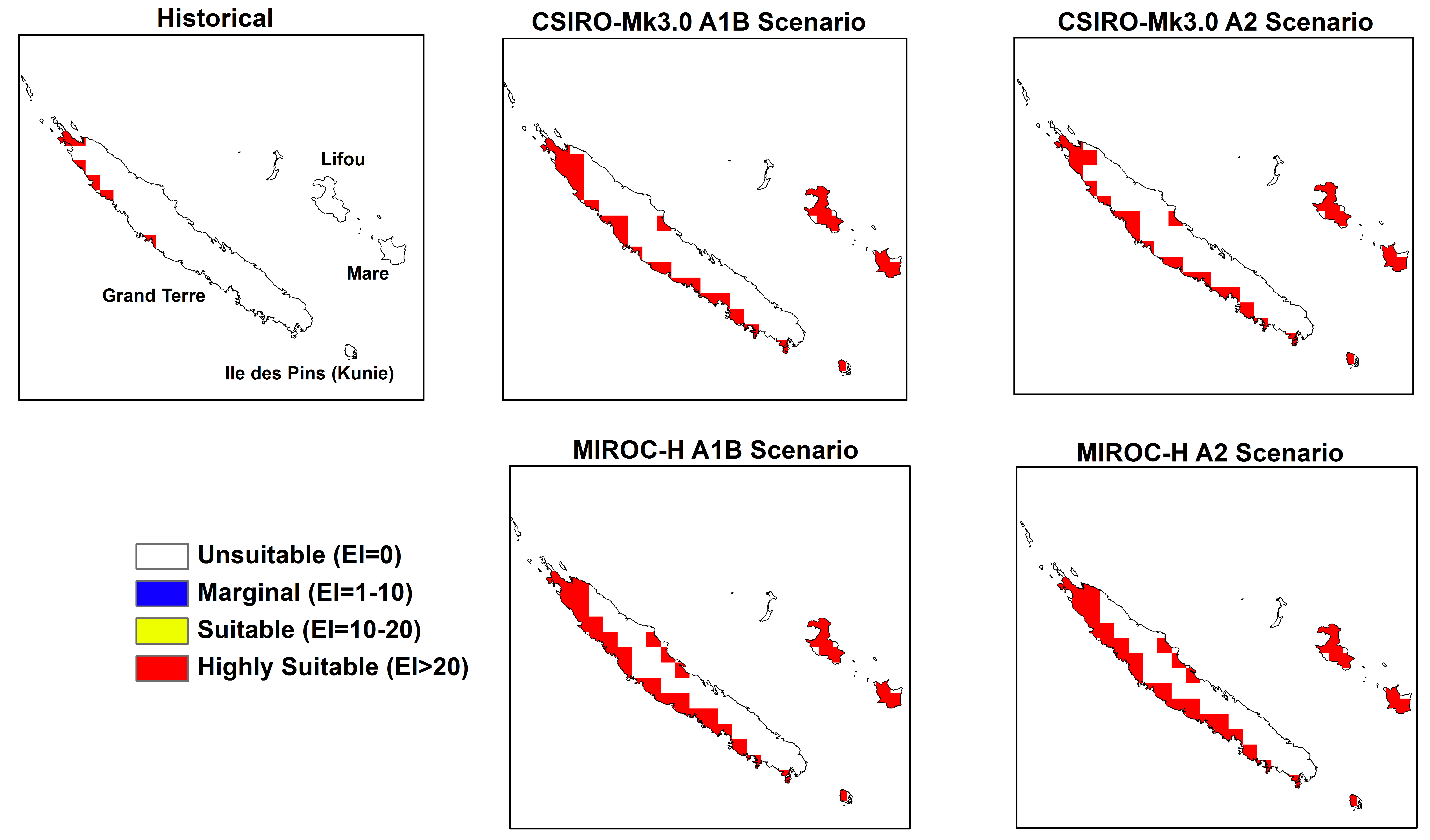

Supplement: Supplementary file 3 — Figure S3. The climate (EI) for M. peltata in New Caledonia for 2030. [file ECE3-6-742-s003.tif]

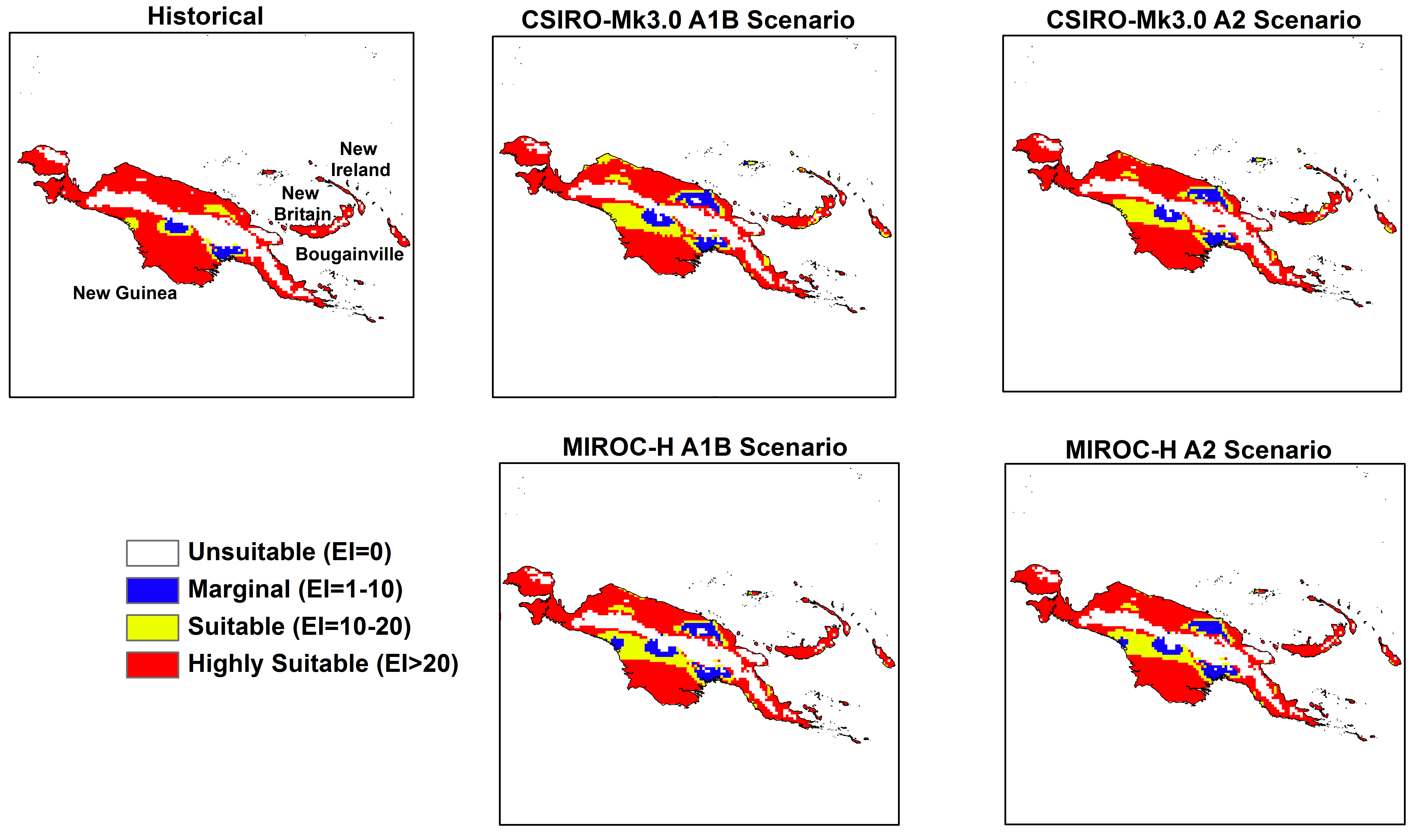

Supplement: Supplementary file 4 — Figure S4. The climate (EI) for M. peltata in Papua New Guinea for 2030. [file ECE3-6-742-s004.tif]

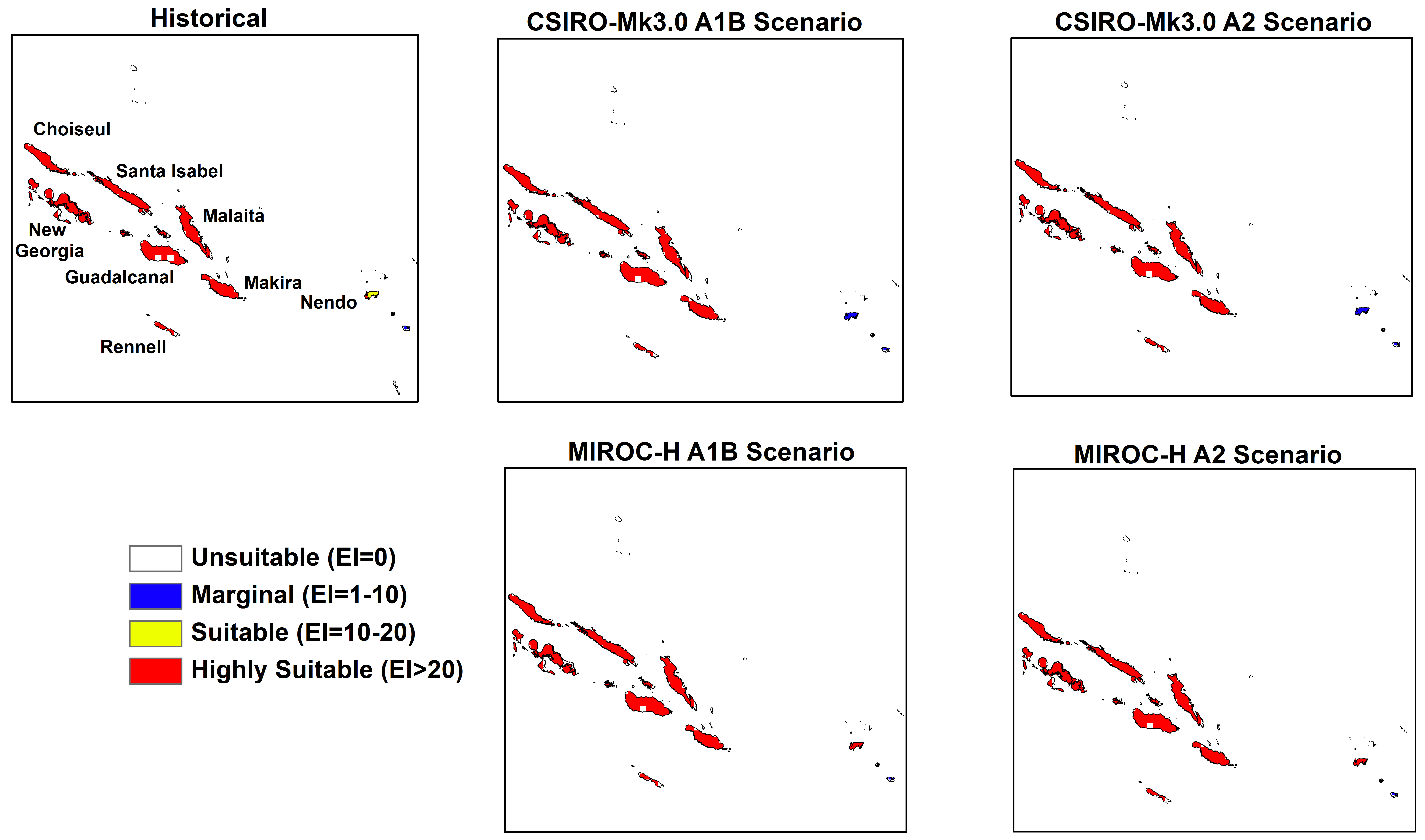

Supplement: Supplementary file 5 — Figure S5. The climate (EI) for M. peltata in Solomon Islands for 2030. [file ECE3-6-742-s005.tif]

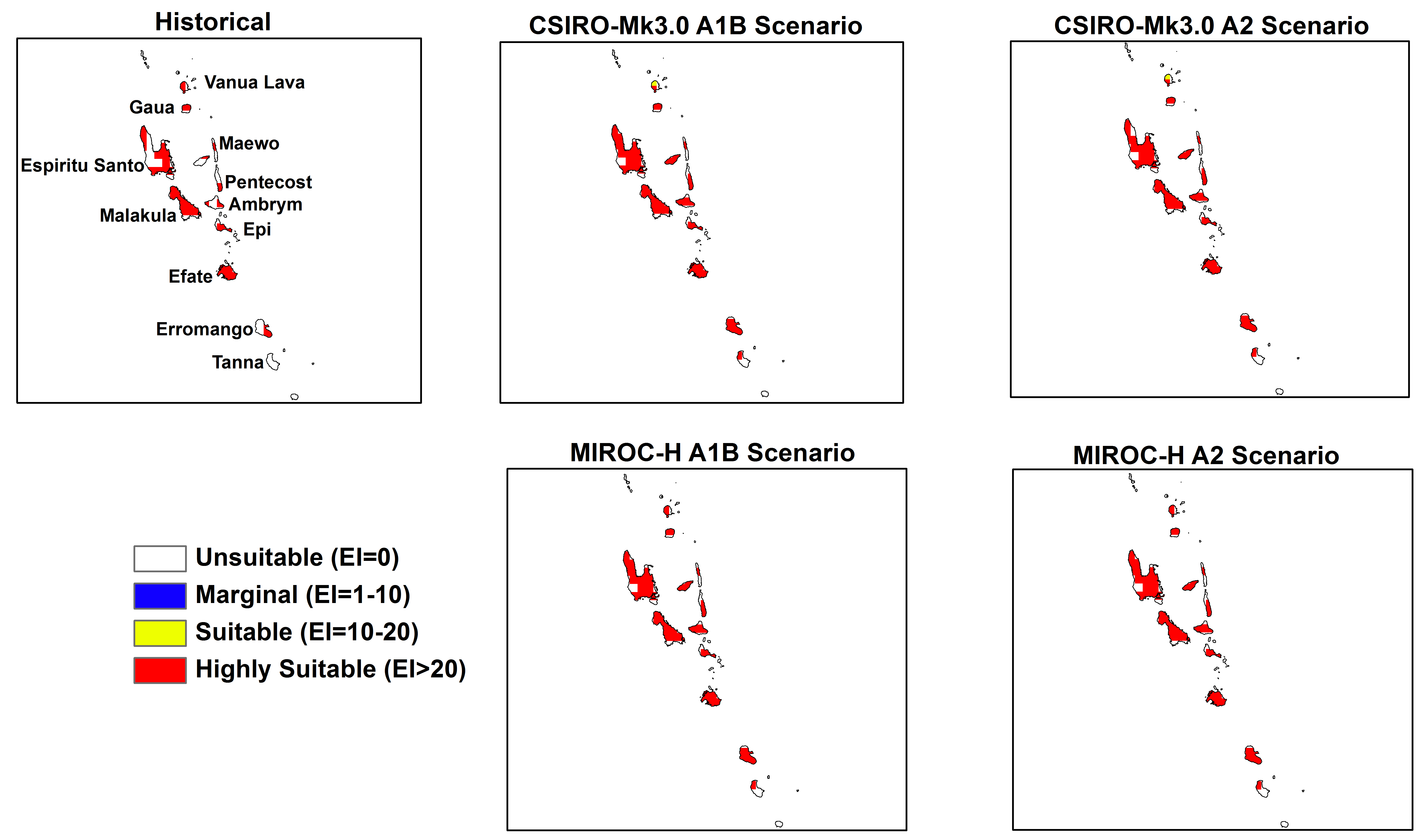

Supplement: Supplementary file 6 — Figure S6. The climate (EI) for M. peltata in Vanuatu for 2030. [file ECE3-6-742-s006.tif]
